# Lateral Abdominal Wall Hematoma Mimicking Aortic Dissection Presentation: A Case Report

**DOI:** 10.7759/cureus.54717

**Published:** 2024-02-22

**Authors:** Babiker A Eltahir, Mohamed E Abdelhameed, Ward E Abdulla Ghaleb

**Affiliations:** 1 Department of Emergency, NMC Royal Hospital, Khalifa City, Abu Dhabi, ARE; 2 Department of Emergency Medicine, Ibrahim Malik Teaching Hospital, Khartoum, SDN

**Keywords:** acute abdomen, aortic dissection, spontaneous hemorrhage, conservative management, lateral wall hematoma

## Abstract

Lateral abdominal wall hematoma is a rare clinical entity but a great mimicker of other diseases' clinical presentations. In this case report, we present a 42-year-old male patient with a constellation of signs and symptoms that were mistaken for aortic dissection before the lateral abdominal wall hematoma diagnosis was confirmed with computed tomography (CT) imaging. Uncontrolled hypertension and persistent cough were most likely predisposing factors; the patient was managed conservatively and discharged in a stable condition.

## Introduction

Lateral abdominal wall hematoma is a rare re-occurrence. It is located within the muscle structures that can be contained by the facia or diffuse onto the peritoneal or retroperitoneal space [1,2]. The classic presentation is acute abdominal pain with a tender abdominal mass [1,3]. It can mimic different abdominal pathologies such as appendicitis, diverticulitis, perforated ulcer, and incarcerated hernia, as well as genitourinary and gynecological emergencies [1]. This case report describes the unique presentation of the lateral abdominal wall hematoma that masqueraded as an aortic dissection (AD), a life-threatening emergency.

## Case presentation

The patient is a 42-year-old male with a known diagnosed case of diabetes, hypertension, and coronary artery disease with a recent coronary stenting four months back. The patient is a chronic smoker and non-compliant with his medication. He presented to the emergency room with sudden onset severe left-sided chest pain and left-sided lateral abdominal pain radiating to the left shoulder and the back. The pain started six hours ago with the maximal intensity at onset and was rated 8/10; it is described as sharp, worsens with movement and breathing, and does not improve with analgesic (diclofenac) received in outpatient settings. The patient had a cough for four days, attributing it to smoking, but no vomiting or any other associated symptoms. The patient denies trauma, being on any medications (antiplatelets and anticoagulation), illicit drugs, and coagulation disorder.

On physical examination, the patient looks unwell, in pain, and profusely diaphoretic. Vitals are heart rate is 107 beats per minute; blood pressure is 220/173; respiratory rate is 22 breaths per minute; SpO2 is 100% on room air; and temperature is 36.9 degrees Celsius. The patient is awake, alert, and oriented. Chest air enters bilaterally with few scattered crackles; there is no murmur. The abdomen tenderness is localized to the left lateral side of the abdomen, and the rest of the abdomen is soft. There are no bruises, palpable masses, rashes, or signs of trauma; there are no pulse deficits. There is a difference of 15 mmHg in systolic blood pressure (SBP) and 10 mmHg in diastolic blood pressure (DBP) between the bilateral upper arms.

A differential diagnosis was broadly made to rule out life-threatening emergencies such as ST-elevation myocardial infarction (STEMI), AD, pulmonary embolism, pneumothorax, peptic ulcer disease (PUD) with perforation, and pancreatitis.

**Figure 1:**
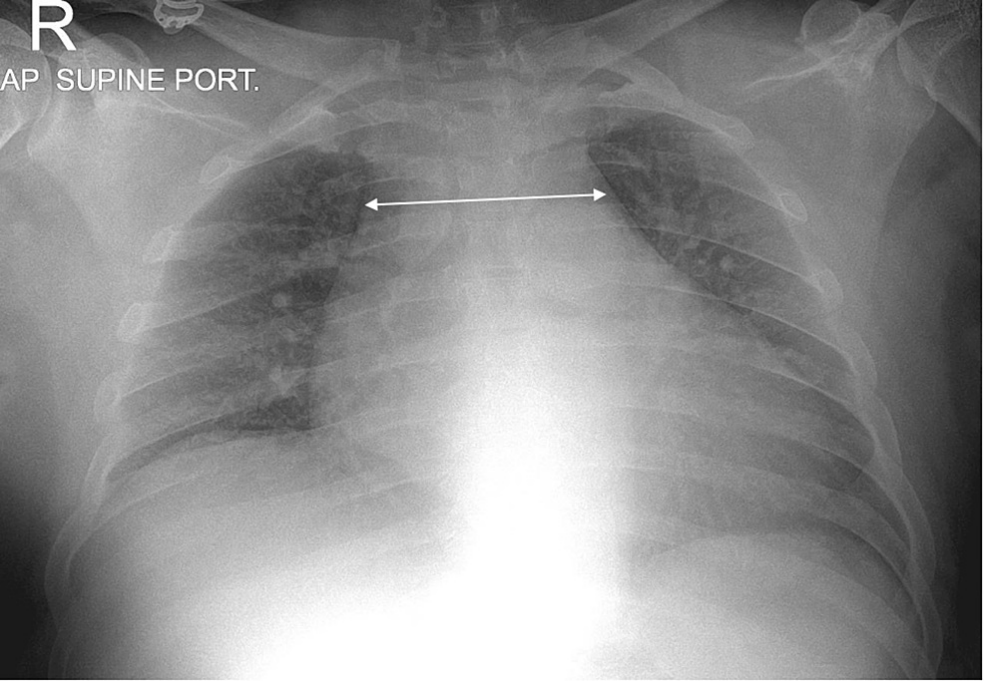
Chest X-ray with prominent mediastinum

An ECG showed no STEMI with ST-t wave changes. The chest X-ray showed a prominent mediastinum with no pneumothorax (Figure 1).

A laboratory investigation was requested, which revealed a normal coagulation screen (International Normalized Ratio (INR): 1.31, prothrombin time (PT): 18 sec, partial thromboplastin time (PTT): 36 sec) and a mild elevation of cardiac enzymes (troponin 0.06 ng/ml; reference range (RR) <0.05 ng/ml and B type natriuretic peptide 253 pg/ml; RR > 100 pg/ml has a threshold for heart failure).

**Figure 2:**
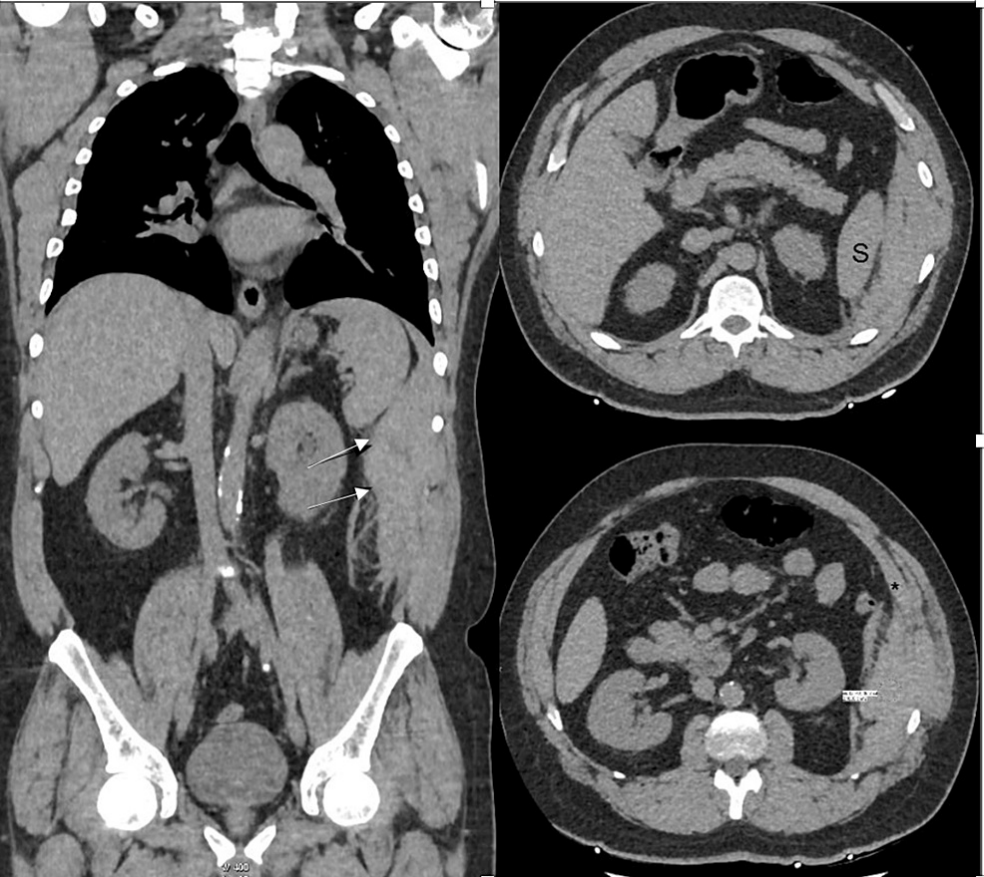
Acute hematoma along the left lateral abdominal wall situated deep to the transversus abdominis muscle, measuring 20 cm in maximal diameter with no active bleeder

Fentanyl was provided with no relief; the patient is still in severe pain and profusely diaphoretic. A CT angiogram was requested to rule out AD and abdominal AD/aneurysm, which showed extraperitoneal acute hematoma along the left lateral abdominal wall, situated deep to the transversus abdominis muscle, measuring 20 cm in maximal diameter with no active bleeding (Figure 2).

The case was with the surgeon and cardiologist, and the patient was admitted for conservative management of his lateral abdominal wall hematoma and heart failure management. After two days, the patient was discharged in stable condition, and a follow-up was arranged with the concerns department. The patient had a follow-up three weeks later with complete symptom resolution.

## Discussion

Lateral abdominal wall hematoma is a subtype of the anterior abdominal wall hematoma, which is rare with unknown prevalence compared to the rectus sheath hematoma, which occurs in around 1.8% of cases [1,4]. It happens due to arterial disruption or muscle layer injuries. The anterior abdominal wall layers consist of the rectus, external oblique, internal oblique, and transverse muscle on each side. These muscles are supplied by deep inferior epigastric arteries, superior epigastric arteries, and deep circumflex arteries [2].

Abdominal wall hematoma has three grades: grade one is an intramuscular hematoma, grade two is an intramuscular hematoma with blood between the muscles and the transversalis fascia, and grade three hematoma may or may not involve the muscle and blood seen between the transversalis fascia and the peritoneum and pre-vesical space, which dictates the management plan [5].

Commonly observed signs and symptoms of abdominal hematoma encompass abdominal pain, the presence of an abdominal mass, ecchymosis of the abdominal wall, nausea, vomiting, tachycardia, peritoneal irritation, abdominal distension, and cramping [6]. Our patient presented with AD features such as abrupt onset sharp pain, which is considered the most common descriptor of pain in around 65% of AD cases, with severe maximal intensity at onset (90%), radiating to the back (85-95%), pain above and below the diaphragm, which has a likelihood ratio (LR) of 7.6% [7-9], high blood pressure (70%) [10], non-specific ST segment changes on ECG (40%) in AD cases [11], widened mediastinum (60-90% of cases and associated with an +LR 1.1-3.4), loss of aortic knob on the chest X-ray [12,13], troponin (26.8% of AD cases), and B type natriuretic peptide elevation [14,15].

Abdominal wall hematoma can arise from various causes, both traumatic and nontraumatic. Further, it is divided into four major groups. Group one is the weakness of the blood vessel due to uncontrolled hypertension, arteriosclerosis, and inflammatory disease; group two is decreased muscular resistance from pregnancy or delivery, previous surgery, obesity, aging, and trauma; group three happens because of over-contracting or over-stretching of the muscle due to violent coughing, sneezing, straining, and vomiting; group four arises from coagulation disorders or anticoagulation medications. In the presented case, the patient exhibited two of the documented predisposing factors, namely, a persistent cough and poorly controlled hypertension [1,4].

Notably, the diagnostic approach predominantly involves contrast-enhanced CT with sensitivity and specificity approximating around 100% compared to the ultrasound, with a sensitivity reaching 80% to 90% [3], with CT establishing the diagnosis and providing essential information about the location, size, origin, and extension of the hematoma and the presence of active bleeding, as well as ruling out life-threatening pathology if the initial diagnosis is uncertain [2]. The US can be used during the follow-up period [4].

For most patients with abdominal wall hematoma, conservative treatment is deemed appropriate, with surgical intervention or angiography with embolization reserved for specific scenarios such as hematoma progression, rupture into the peritoneal cavity, or the presence of infection [2].

## Conclusions

In summary, lateral abdominal wall hematoma, though rare, can present with symptoms resembling life-threatening conditions. This case emphasizes the diagnostic complexity faced when a young male initially appeared to have AD but was later confirmed to have a lateral abdominal wall hematoma through CT imaging. Uncontrolled hypertension and a persistent cough were identified as significant predisposing factors. Despite the initial diagnostic challenge, conservative management led to the patient's stable discharge after two days. This underscores the importance of considering lateral abdominal wall hematoma in the differential diagnosis when atypical symptoms are present. Healthcare providers should remain vigilant and employ comprehensive diagnostic approaches to distinguish this rare condition from potentially life-threatening emergencies.
